# Supplementary material for: Progressive relaxation training in patients with breast cancer receiving aromatase inhibitor therapy-randomized controlled trial
Source: PLoS One. 2024 Apr 18;19(4):e0301020. doi: 10.1371/journal.pone.0301020 (PMC11025930; doi:10.1371/journal.pone.0301020)
Supplement: S1 File — (DOCX) [file pone.0301020.s002.docx]

**ARAŞTIRMA PROTOKOLÜ**

**ARAŞTIRMANIN ADI:** Aromataz inhibitörü kullanan meme kanserli hastalarda ilerleyici gevşeme egzersizlerinin etkileri

**ARAŞTIRMANIN AMACI:** Aromataz inhibitörü kullanan meme kanserli hastalarda ilerleyici gevşeme egzersizlerinin; tedavi kaynaklı artraljik ağrılar, yaşam kalitesi ve anksiyete-depresyon durumu üzerine etkilerini incelemek.

**ARAŞTIRMA HAKKINDA GENEL BİLGİ**

Meme kanseri kadın kanserleri içinde en fazla görülen kanserdir. 2018 “IARC” verilerine göre dünyada meme kanseri insidansının 100.000’de 46,3; mortalitenin ise 100.000’de 13 olduğu belirtilmiştir.^1^

Aromataz inhibitörlerinin yüksek riskli postmenopozal kadınlarda meme kanseri insidansını etkili bir şekilde düşürdüğü bilinmektedir^2^. Hormon reseptörü pozitif, posmenomopzal meme kanserli hastalarda tedavi yöntemlerinden biri olarak aromataz inhibitörleri kullanılmaktadır. Aromataz inhibitörleri, androjenin östrojene dönüşmesini sağlayan aromataz enzimini engelleyip östrojen miktarını azaltmaktadır. Kan dolaşımında daha az östrojen olması kanser hücresi çoğalmasını engellemektedir.^2,3^

Aromataz inhibitörü kullanan hastalarda muskoloskeletal problemler ve fraktür riskinin arttığı gözlemlenmiştir.^4-6^ Özellikle artralji, aromataz inbitörü alan hastaların neredeyse % 50’sinde gözlemlenebilmektedir.^7^ Bunun dışında aromataz inhibitör alan hastalarda kognitif fonksiyon bozuklukları^8^, yaşam kalitesi düşüşleri^9^, anksiyete ve depresyon^10^, uyku problemleri ve yorgunluk^11^ gibi problemlerde gözlemlenebilmektedir. Bunun gibi sağlık profillerinin, ağrı ve semptomların değerlendirilmesi ve tedavisinin sağlanması, hastaların yaşam kalitesini arttırma açısından önemlidir.

İlerleyici kas gevşeme egzersizleri ilk olarak 1938 yılında Jacobson tarafından tanımlanmıştır.^12^ Daha sonra farklı düzenleme ve güncellemelerle günümüzde de kullanımına devam edilmektedir.^13,14^ Kas gevşemesinin fizyolojik, algısal ve davranışsal olumlu bulguları tanımlanmıştır. Gevşeme için sırtüstü, yarım yatma, yüzüstü ve yan yatma pozisyonları kullanılabilmektedir.^15^

Bu bilgiler doğrultusunda planlanan bu çalışmada ki amacımız; aromataz inhibitörü kullanan meme kanserli hastalarda ilerleyici gevşeme egzersizlerinin etkilerini incelemektir.

**HİPOTEZLER**

**H1:** Aromataz inhibitörü kullanan meme kanserli hastalarda ilerleyici gevşeme egzersizleri; tedavi kaynaklı artraljik ağrılar, yaşam kalitesi ve anksiyete-depresyon durumu düzeyi üzerine etkilidir.

**H0:** Aromataz inhibitörü kullanan meme kanserli hastalarda ilerleyici gevşeme egzersizleri; tedavi kaynaklı artraljik ağrılar, yaşam kalitesi ve anksiyete-depresyon durumu düzeyi üzerine etkili değildir.

**MATERYAL VE METOD**

**Çalışma popülasyonu ve yöntemi:** Çalışma randomize kontrollü olarak yapılacak olup, kurum izni alınmış olan (Ek-1) Gayrettepe Florence Nightingale Hastanesi’nde yürütülecektir. 1 Ocak 2017-1 Temmuz 2020 tarihleri arasında hormon reseptör pozitif olup aromataz inhibitörü kullanan hastalar, Doç. Dr. Çetin Ordu’nun klinikte takip ettiği hasta kayıtlarını içeren arşivden taranıp bulunacaktır. Telefonla ulaşılan hastalardan en az hafif artralji (Kısa ağrı envanterine göre ağrı skoru ≥3 olanlar) tanımlayan hastalar saptanıp değerlendirilmek üzere kliniğe çağırılacaktır. Hastalardan yazılı onam (Ek-2) alındıktan sonra, hastalar bilgilendirme grubu veya gevşeme egzersiz grubuna dahil edilecektir. Örneklem büyüklüğünü hesaplamak için referans makaleden^16^ alınan standart sapma ve güven aralığı verileriyle yapılan güç analizinde (%80 güç ve %5 tip 1 hata) egzersiz grubu ve kontrol grubuna 44’er hasta alınması planlandı. Bireylerin gruplara atanması için gereken randomizasyon işlemi, çevirim içi “random allocation software program” kullanılarak yapılacak. Değerlendirmeler ilk görüşmede ve 6 haftalık egzersiz eğitimi veya bilgilendirme sonrası yapılacaktır. Çalışmaya başlamadan önce hastaların demografik ve hastalık ile ilgili bilgileri değerlendirme formuna (Ek-3) kaydedilecektir.

Dahil edilme kriteleri:

1. Evre 1-3 arası meme kanseri tanısı
2. 6 aydan fazla aromataz inhibitör kullanıyor olmak
3. Kısa Ağrı Envanterine göre ağrı skoru ≥3 puan almış olmak
4. 30-70 yaş aralığında olmak

Dışlama kriterleri:

1. İletişim probleminin olması
2. Ağrıya sebep olacak nörolojik veya ortopedik problemlerin varlığı
3. İleri dönem lenfödem tanısının varlığı

**Egzersiz** **Grubu:** Bu grupta bulunan hastalar için, haftada 1 gün hastanede süpervize ve haftada 3 gün ev programı şeklinde, ilerleyici gevşeme egzersizleri programı 6 hafta boyunca verilecektir. Hastalar 6-8 kişilik gruplar halinde alınacaktır. Hastaların ev programlarını takip etmek amacıyla hastalar telefonla aranacak ve bir çizelge hazırlanacaktır (Ek-4). Hastaların ilerleyici gevşeme egzersizleri, onkolojik rehabilitasyon alanında 7 yıllık deneyimi olan Uzm. Fizyoterapist Umut Bahçacı tarafından verilecektir.

İlerleyici gevşeme egzersizleri Jacobson ve ark.^12^ tarafından 1938 yılında tanımlanmış olup daha sonra bazı çalışmalarla güncellenmiştir.^15^ Çalışma alanı iyi havalandırılmış ve hastaların rahat edeceği bir ortam olacaktır. Çalışmamızda hastalar önce rahat edebilecekleri koltuklarda uzun oturma pozisyonuna alınacaktır. Daha sonra hastalara sırasıyla aşağıdaki talimatlar verilecektir.

- Ellerinizi yumruk yapın, önkolunuzu kasın ve hareketi bırakın
- Ellerinizi yumruk yapın, dirseğinizi koltuğa doğru itin
- Dirseklerinizi bükün
- Omuzlarınızı geriye doğru itin
- Dizinizi aşağı doğru basıp, ayak parmaklarınızı kendinize doğru çekin
- Dizlerinizi kendinize doğru çekip, ayaklarınızı aşağı doğru itin
- Kalçanızı sıkın
- Başınızı arkaya doğru itin
- Kaşlarınızı kaldırın
- Burnunuzda kırışıklıklar yapın
- Dişlerinizi sıkın
- Çenenizi aşağı doğru itin
- Gözlerinizi kapatın ve iyi şeyler düşünün.

Egzersizler 5 saniye kasma 20 saniye gevşeme şeklinde uygulanacaktır. Egzersizler arasında zaman zaman solunum çalışması yapılacak olup gevşemenin etkinliği arttırılacaktır. Egzersizler sırasında stres oluşturacak herhangi bir etkenin olmaması için gerekli önlemler alınacaktır.

**Kontrol Grubu:** Bu gruptaki hastalarımıza ağrı ve tedavisi ile ilgili bilgilendirmeler yapılacak olup, ev içi ve çevresel önlemler konusunda bilgi verilecektir. Hastaların stresli yaşam koşullarından uzaklaşmaları ve ev içinde kendilerine gevşeyebilecekleri zaman ayırmaları konusunda önerilerde bulunulacaktır. Çalışma sonunda egzersiz etkililiği sağlandığı takdirde, bu hastalarımıza da süpervize egzersiz verilecektir.

**Çalışmada kullanılacak değerlendirmeler**:

1. **Kısa ağrı envanteri:** Türkçe validasyonu yapılmış^17^, 9 sorudan oluşan, ağrının yerini, şiddetini, özellikle son 24 saatlik aktivitelerle birlikte ağrı durumunu değerlendiren bir ölçektir. Kanser hastalarında sık kullanılan kısa ağrı envanterinin için geçerlik güvenirlik çalışması yapılmıştır.^18^ Bu ölçekte 3-4 arası skorlar hafif, 5-7 arası skorlar orta, 8-10 arası skorlar şiddetli ağrı olarak tanımlanır. Çalışmaya ağrı skorları 3 ve üzeri olan hastalar dahil edilecek olup, sonraki değerlendirmelerde de bu ölçek kullanılacaktır.
2. **Functional Assessment of Chronic Illness Therapy- Breast (FACT-B):** Meme kanserli hastalarda çok yönlü yaşam kalitesini değerlendirmek için hazırlanmış; hastaların kendi durumlarını değerlendirdikleri 27 maddelik genel ve 10 maddelik meme kanserine yönelik bir ölçektir. Ankette fiziksel, sosyal, duygusal, işlevsel ve diğer endişe durumunun değerlendirdiği 5 alt skaladan bulunmaktadır. Hastalar son 7 gün içerisinde belirli bir ifadenin kendileri için ne kadar geçerli olduğunu 0; hiç, 1; az, 2; biraz, 3; oldukça, 4; çok fazla ölçütlerini içeren 5 puanlık bir skala ile belirlemektedir. Yüksek puanlar yaşam kalitesinin yüksek olduğunu, düşük puanlar ise yaşam kalitesinin düştüğünü ifade etmektedir. Türkçe versiyonuna ulaşmak ve kullanmak için [www.facit.org](http://www.facit.org) adresinden gerekli izinler alınmıştır.^19^
3. **Hastane Anksiyete ve Depresyon (HAD) skalası:** 14 sorudan oluşan ve Türkçe geçerlik ve güvenirlik çalışması yapılmış bir ölçektir.^20^ Bu sorulardan 7’si anksiyeteyi, 7’si depresyonu değerlendirmektedir. Likert tipi ölçüm yapılmaktadır. Anksiyete alt ölçeği için kesme puanı 10/11, depresyon alt ölçeği için ise 7/8’ dir.. Buna göre bu puanların üzerinde alanlar risk altında olarak değerlendirilir.

**İstatistiksel Analiz:**

İstatistiksel değerlendirmede SPSS version 16.0 (Copyright © SPSS Inc., 1988-2007. All Rights reserved, Licensed to: TEAM EQX 6th. Birthday 1337) programı kullanılacaktır. Verilerin normal veri dağılımlarına uyup uymadığı, Kolmogorov-Simirnov testi ile bakılacaktır. Hasta ve kontrol grubununda öncesi ve sonrası değerlendirmeleri dağılım özelliklerine göre eşleştirilmiş örneklem t testi veya Wilcoxon testi ile bakılacak, tedavi sonrası iki grup arasında karşılaştırmalar dağılım özelliklerine göre bağımsız örneklem t testi veya Mann-Whitney U testi ile değerlendirilecektir.

**Referanslar**

1. Breast, Source: GLOBACAN. 2019. <https://gco.iarc.fr/today/data/factsheets/cancers/20-Breast-fact-sheet.pdf>. Accessed 09.03.2018.

2. Cuzick J, Sestak I, Forbes JF, et al. Anastrozole for prevention of breast cancer in high-risk postmenopausal women (IBIS-II): an international, double-blind, randomised placebo-controlled trial. *The Lancet.* 2014;383(9922):1041-1048.

3. Group EBCTC. Aromatase inhibitors versus tamoxifen in early breast cancer: patient-level meta-analysis of the randomised trials. *The Lancet.* 2015;386(10001):1341-1352.

4. Sabel MS. Chapter 17 - Principles of Adjuvant Hormonal Therapy. In: Sabel MS, ed. *Essentials of Breast Surgery.* Mosby; 2009:267-278.

5. The A. Anastrozole alone or in combination with tamoxifen versus tamoxifen alone for adjuvant treatment of postmenopausal women with early breast cancer: first results of the ATAC randomised trial. *The Lancet.* 2002;359(9324):2131-2139.

6. Mincey BA, Duh MS, Thomas SK, et al. Risk of cancer treatment—associated bone loss and fractures among women with breast cancer receiving aromatase inhibitors. *Clinical breast cancer.* 2006;7(2):127-132.

7. Crew KD, Greenlee H, Capodice J, et al. Prevalence of joint symptoms in postmenopausal women taking aromatase inhibitors for early-stage breast cancer. *Journal of Clinical Oncology.* 2007;25(25):3877-3883.

8. Bender CM, Sereika SM, Brufsky AM, et al. Memory impairments with adjuvant anastrozole versus tamoxifen in women with early-stage breast cancer. *Menopause (New York, NY).* 2007;14(6):995.

9. Cella D, Fallowfield L, Barker P, Cuzick J, Locker G, Howell A. Quality of life of postmenopausal women in the ATAC (“Arimidex”, tamoxifen, alone or in combination) trial after completion of 5 years' adjuvant treatment for early breast cancer. *Breast cancer research and treatment.* 2006;100(3):273-284.

10. Breckenridge LM, Bruns GL, Todd BL, Feuerstein M. Cognitive limitations associated with tamoxifen and aromatase inhibitors in employed breast cancer survivors. *Psycho‐Oncology.* 2012;21(1):43-53.

11. So WK, Marsh G, Ling W, et al. The symptom cluster of fatigue, pain, anxiety, and depression and the effect on the quality of life of women receiving treatment for breast cancer: a multicenter study. Paper presented at: Oncology nursing forum2009.

12. Jacobson E. Progressive muscle relaxation. *Interview Behaviour" Journal of Abnormal Psy-University of Chicago Piess, Chicago chology.* 1938;75(1):18.

13. Bell JA, Saltikov JB. Mitchell's relaxation technique: Is it effective? *Physiotherapy.* 2000;86(9):473-478.

14. Bernstein DA, CARLSON CR, SCHMIDT JE. Progressive relaxation. *Stress Management.* 1973:88.

15. Otman A, Köse N. Egzersiz tedavisinde temel prensipler ve yöntemler. *Meteksan AŞ.* 2006:21-51.

16. GALANTINO, Mary Lou, et al. Impact of yoga on functional outcomes in breast cancer survivors with aromatase inhibitor–associated arthralgias. Integrative Cancer Therapies, 2012, 11.4: 313-320.

17. Dicle A, Karayurt Ö, Dirimese EJPMN. Validation of the Turkish version of the Brief Pain Inventory in surgery patients. 2009;10(2):107-113. e102.

18. Cleeland C, Ryan KJA, Academy of Medicine, Singapore. Pain assessment: global use of the Brief Pain Inventory. 1994.

19. Fallowfield LJ, Leaity SK, Howell A, Benson S, Cella DJBcr, treatment. Assessment of quality of life in women undergoing hormonal therapy for breast cancer: validation of an endocrine symptom subscale for the FACT‐B. 1999;55(2):187-197.

20. Aydemir OJTPD. Hastane anksiyete ve depresyon olcegi Turkce formunun gecerlilik ve guvenilirligi. 1997;8:187-280.

**Ek 1.**

27.03.2019

**İstanbul Demiroğlu Bilim Üniversitesi**

**Klinik Araştırmalar Etik Kurulu Başkanlığı’na**

Uzm. Fizyoterapist Umut Bahçacı’nın kurumumuzun Medikal Onkloji Kliniği’nde “**Aromataz inhibitör kullanan meme kanserli hastalarda ilerleyici gevşeme egzersizlerinin etkileri**” adlı girişimsel olmayan klinik araştırmasını yapması uygun görülmüştür.

Gayrettepe Florence Nightingale Hastanesi

Tıbbi direktör

Dr. Özay Ünal

**Ek 2.**

**ARAŞTIRMA AMAÇLI ÇALIŞMA İÇİN BİLGİLENDİRİLMİŞ GÖNÜLLÜ ONAM FORMU**

Araştırmamızın ismi ‘Aromataz inhibitörü kullanan meme kanserli hastalarda ilerleyici gevşeme egzersizlerinin etkileri’dir. Bu bilgileri okuyup anladıktan sonra araştırmaya katılmak isterseniz formu imzalayınız.

Eğer çalışmaya katılmayı kabul ederseniz, sorumlu araştırmacı Doç. Dr. Zeynep Erdoğan İyigün tarafından demografik bilgileriniz alınıp ağrı değerlendirmeniz yapılacaktır. Daha sonra yaşam kalitesi, anksiyete depresyon durumunuzu ölçmek için kullanılan değerlendirmeler uygulanacaktır. Değerlendirme sonucunda Doç. Dr. Zeynep Erdoğan İyigün tarafından uygun bulunduğunuz takdirde Uzm. Fzt. Umut Bahçacı süpervizörlüğünde ilerleyici gevşeme egzersizleri programına dahil edileceksiniz. Gevşeme egzersizleri 6 hafta sürecek olup bu süre sonunda ikinci değerlendirmeleriniz yapılacaktır. Değerlendirme kayıtlarınız kimliğiniz belirtilmeden sağlık alanında öğrenim gören öğrencilerin eğitiminde veya bilimsel nitelikte yayınlarda kullanılabilir. Bunun dışında bu kayıtlar kullanılmayacak ve başkalarına verilmeyecektir.

Çalışmada; gönüllülerin isimleri kullanılmayacak olup sadece sonuçları istatistiksel olarak bilimsel yazı şeklinde yayınlanacaktır. Sağlık otoriteleri, Bakanlık, Etik Kurul gerektiğinde gönüllülerin kayıtlarına ulaşabilir ancak bu bilgiler gizli tutulacaktır. Gönüllüler isterlerse çalışmanın sonuçları hakkında bilgilendirileceklerdir, ayrıca istedikleri anda çalışmadan ayrılma hakkına sahiptirler. Gönüllülere bu çalışma için çalışmadan ayrılsalar dahi herhangi bir tazminat ve ek bir ödeme yapılmayacaktır. Çalışma sırasında ve sonrasında ek bir tedavi uygulanmayacaktır.

Değerlendirmeler sırasında oluşabilecek riskler: çalışma kapsamında yapılacak olan değerlendirmeler ve uygulanacak program, herhangi bir risk içermemektedir.

**Katılımcının Beyanı**

Sayın Doç. Dr. Zeynep Erdoğan İyigün tarafından “Aromataz inhibitörü kullanan meme kanserli hastalarda ilerleyici gevşeme egzersizlerinin etkileri”nin araştırılması amacıyla bir araştırma yapılacağı belirtilerek, bu araştırma ile ilgili yukarıdaki bilgiler bana aktarıldı. Bu bilgilendirmelerden sonra böyle bir araştırmaya ‘katılımcı’ olarak davet edildim.

Eğer bu araştırmaya katılırsam doktor ile aramda kalması gereken bana ait bilgilerin gizliliğine bu araştırma sırasında da büyük özen ve saygı ile yaklaşılacağına inanıyorum. Araştırma sonuçlarının eğitim ve bilimsel amaçlarla kullanımı sırasında kişisel bilgilerimin ihtimamla korunacağı konusunda bana yeterli güven verildi.

Bilgilendirilmiş Gönüllü Onam Formundaki tüm açıklamaları okudum. Bana yukarıda konusu ve amacı anlatılan araştırma ile ilgili yazılı ve sözlü açıklama aşağıda adı belirtilen araştırmacı tarafından yapıldı. Araştırmaya gönüllü olarak katıldığımı, istediğim zaman gerekçeli ya da gerekçesiz olarak araştırmadan ayrılabileceğimi ve kendi isteğime bakılmaksızın araştırmacı tarafından araştırma dışı bırakılabileceğimi biliyorum.

Bana yapılan tüm açıklamaları ayrıntılarıyla anlamış bulunmaktayım. Kendi başıma adı geçen bu araştırmada gönüllü olarak yer alma kararını aldım. Bu konuda yapılan daveti büyük bir memnuniyet ve gönüllülük içerisinde kabul ediyorum.

İmzalı bu form kâğıdının bir kopyası bana verilecektir.

**Söz konusu araştırmaya, hiçbir baskı ve zorlama olmaksızın kendi rızamla katılmayı**

**Kabul ediyorum Kabul etmiyorum**

**Bu çalışma için alınan bilgilerin ileride yapılacak başka bir çalışmada kullanılmasını**

**Kabul ediyorum Kabul etmiyorum**

**Katılımcı**

Adı, soyadı:

Adres:

Tel:

İmza:

**Görüşme tanığı**

Adı, soyadı:

Adres:

Tel.

İmza:

**Katılımcı ile görüşen çalışmacı**

Adı, soyadı:

Adres:

Tel.

İmza:

Tarih: Tel:

**Ek-3.**

**Değerlendirme Formu**

AROMATAZ İNHİBİTÖRÜ KULLANAN MEME CA HASTALARINDA ARTİKÜLER TUTULUMLARIN DEĞERLENDİRİLMESİ

AD-SOYAD:

YAŞ:

BOY: KİLO: VKI:

BEL ÇEVRESİ:

MEDENİ DURUM:

MESLEK:

EĞİTİM DURUMU:

DOMİNANT EKSTREMİTE:

TANI TARİHİ:

OPERASYON ZAMANI:

OPERASYON TARAFI:

OPERASYON TİPİ:

AKSİLLER DİSEKSİYON:

KT (AJANLAR):

TAKSAN

TMX

RT:

AROMOTAZ İNH. ADI:

KULLANDIĞI SÜRE:

ÇAY: KAHVE:

ALKOL: SİGARA:
