# Supplementary material for: Progressive relaxation training in patients with breast cancer receiving aromatase inhibitor therapy-randomized controlled trial
Source: PLoS One. 2024 Apr 18;19(4):e0301020. doi: 10.1371/journal.pone.0301020 (PMC11025930; doi:10.1371/journal.pone.0301020)
Supplement: S2 File — (DOCX) [file pone.0301020.s003.docx]

**RESEARCH PROTOCOL**

**TITLE OF THE STUDY:** The effects of progressive relaxation exercises in breast cancer patients using aromatase inhibitors

**AIM OF THE RESEARCH:** To investigate the effects of progressive relaxation exercises on treatment-induced arthralgic pain, quality of life and anxiety-depression status in breast cancer patients using aromatase inhibitors.

**GENERAL INFORMATION ABOUT THE RESEARCH**

Breast cancer is the most common cancer among female cancers. According to 2018 "IARC" data, the incidence of breast cancer in the world is 46.3 per 100,000 and mortality is 13 per 100,000.^1^

Aromatase inhibitors are known to effectively reduce the incidence of breast cancer in high-risk postmenopausal women^2^. Aromatase inhibitors are used as one of the treatment modalities in patients with hormone receptor positive posmenomopause breast cancer. Aromatase inhibitors reduce the amount of estrogen by inhibiting the aromatase enzyme that converts androgen into estrogen. Less estrogen in the bloodstream prevents cancer cell proliferation.^2,3^

An increased risk of musculoskeletal problems and fractures has been observed in patients taking aromatase inhibitors.^4-6^ Especially arthralgia can be observed in almost 50% of patients taking aromatase inhibitors.^7^ In addition, cognitive dysfunction in patients taking aromatase inhibitors^8^decreases in quality of life^9^anxiety and depression^10^sleep problems and fatigue^11^ problems such as pain and symptoms can be observed. Evaluation and treatment of such health profiles, pain and symptoms are important in terms of improving the quality of life of patients.

Progressive muscle relaxation exercises were first described by Jacobson in 1938.^12^ Afterwards, it continues to be used today with different arrangements and updates.^13,14^ Physiological, perceptual and behavioural positive findings of muscle relaxation have been defined. Supine, half lying, prone and side lying positions can be used for relaxation.^15^

The aim of this study, which was planned in line with this information, was to investigate the effects of progressive relaxation exercises in breast cancer patients using aromatase inhibitors.

**HYPOTHESIS**

**H1:** Progressive relaxation exercises are effective on treatment-induced arthralgic pain, quality of life and anxiety-depression level in breast cancer patients using aromatase inhibitors.

**H0:** Progressive relaxation exercises are not effective on treatment-induced arthralgic pain, quality of life and anxiety-depression level in breast cancer patients using aromatase inhibitors .

**MATERIAL AND METHOD**

**Study population and method: The** study will be a randomised controlled trial and will be conducted at Gayrettepe Florence Nightingale Hospital, for which institutional approval has been obtained (Appendix-1). Between 1 January 2017 and 1 July 2020, patients who are hormone receptor positive and use aromatase inhibitors will be scanned from the archive containing the patient records followed by Assoc. Prof. Dr. Çetin Ordu in the clinic. Among the patients contacted by telephone, patients who describe at least mild arthralgia (pain score ≥3 according to the short pain inventory) will be identified and invited to the clinic for evaluation. After written informed consent (Appendix-2) is obtained from the patients, they will be included in the information group or relaxation exercise group. To calculate the sample size, the reference article^16^ In the power analysis performed with the standard deviation and confidence interval data (80% power and 5% type 1 error), 44 patients were planned to be included in the exercise group and control group. The randomisation process required for the assignment of individuals to the groups will be performed using the online random allocation software program. Assessments will be made at the first interview and after 6 weeks of exercise training or counselling. Before starting the study, demographic and disease-related information of the patients will be recorded in the evaluation form (Appendix-3).

Inclusion criteria:

- Diagnosis of stage 1-3 breast cancer
- Being on aromatase inhibitors for more than 6 months
- Having a pain score ≥3 points according to the Short Pain Inventory
- To be between the ages of 30-70

Exclusion criteria:

- Communication problems
- Presence of neurological or orthopaedic problems that may cause pain
- Presence of a diagnosis of advanced lymphoedema

**Exercise Group:** For patients in this group, a programme of progressive relaxation exercises will be given for 6 weeks as supervised 1 day a week in the hospital and 3 days a week home programme. Patients will be recruited in groups of 6-8 people. Patients will be telephoned and a chart will be prepared to follow the patients' home programmes (Appendix-4). Progressive relaxation exercises of the patients will be performed by Specialist Physiotherapist Umut Bahçacı, who has 7 years of experience in the field of oncological rehabilitation. Physiotherapist Umut Bahçacı who has 7 years of experience in oncological rehabilitation.

Progressive relaxation exercises were described by Jacobson et al.^12^ Jacobson et al. in 1938 and later updated by some studies.^15^ The study area will be well ventilated and the patients will be comfortable. In our study, patients will first be placed in a long sitting position in armchairs where they will be comfortable. Then the following instructions will be given to the patients respectively.

- Make your hands into fists, contract your forearm and release the movement
- Make your hands into fists, push your elbow towards the seat
- Bend your elbows
- Push your shoulders back
- Press your knee downwards and pull your toes towards you
- Pull your knees towards you and push your feet downwards
- Squeeze your buttocks
- Push your head backwards
- Raise your eyebrows
- Make wrinkles on your nose
- Grit your teeth
- Push your chin downwards
- Close your eyes and think good thoughts.

Exercises will be applied as 5 seconds contraction and 20 seconds relaxation. Respiratory work will be done from time to time between the exercises and the effectiveness of relaxation will be increased. Necessary precautions will be taken to avoid any stressful factors during the exercises.

**Control Group:** Patients in this group will be informed about pain and its treatment, and information about domestic and environmental precautions will be given. Patients will be advised to get away from stressful living conditions and to allocate time for themselves to relax at home. At the end of the study, if exercise effectiveness is achieved, these patients will also be given supervised exercise.

**Assessments to be used in the study**:

- **Brief pain inventory:** Turkish validated^17^It is a scale consisting of 9 questions that evaluates the location and intensity of pain and pain status, especially with activities in the last 24 hours. A validity and reliability study was conducted for the short pain inventory which is frequently used in cancer patients.^18^ In this scale, scores between 3-4 are defined as mild pain, scores between 5-7 as moderate pain, and scores between 8-10 as severe pain. Patients with pain scores of 3 and above will be included in the study and this scale will be used in subsequent evaluations.
- **Functional Assessment of Chronic Illness Therapy- Breast (FACT-B):** It is a 27-item general and 10-item breast cancer-oriented scale prepared to evaluate the multidimensional quality of life in patients with breast cancer. The questionnaire includes 5 subscales that assess physical, social, emotional, functional and other concerns. Patients determine how valid a certain statement is for them in the last 7 days with a 5-point scale including 0; not at all, 1; a little, 2; a little, 3; quite, 4; very much. High scores indicate a high quality of life, while low scores indicate a decreased quality of life. Necessary permissions were obtained from www.facit.org to access and use the Turkish version.^19^
- **Hospital Anxiety and Depression (HAD) scale: It is a** scale consisting of 14 questions and Turkish validity and reliability study has been performed.^20^ Seven of these questions assess anxiety and seven assess depression. Likert-type measurement is made. The cut-off score for the anxiety subscale is 10/11 and 7/8 for the depression subscale. Accordingly, those above these scores are considered to be at risk.

**Statistical Analysis:**

SPSS version 16.0 (Copyright © SPSS Inc., 1988-2007. All Rights reserved, Licensed to: TEAM EQX 6th. Birthday 1337) programme will be used. The Kolmogorov-Simirnov test will be used to determine whether the data conform to normal data distributions. Pre- and post-treatment evaluations of the patient and control groups will be evaluated by paired sample t test or Wilcoxon test according to the distribution characteristics, and post-treatment comparisons between the two groups will be evaluated by independent sample t test or Mann-Whitney U test according to the distribution characteristics.

**References**

1. Breast, Source: GLOBACAN. 2019. https://gco.iarc.fr/today/data/factsheets/cancers/20-Breast-fact-sheet.pdf. Accessed 09.03.2018.

2. Cuzick J, Sestak I, Forbes JF, et al. Anastrozole for prevention of breast cancer in high-risk postmenopausal women (IBIS-II): an international, double-blind, randomised placebo-controlled trial. *The Lancet.* 2014;383(9922):1041-1048.

3. Group EBCTC. Aromatase inhibitors versus tamoxifen in early breast cancer: patient-level meta-analysis of the randomised trials. *The Lancet.* 2015;386(10001):1341-1352.

4. Sabel MS. Chapter 17 - Principles of Adjuvant Hormonal Therapy. In: Sabel MS, ed. *Essentials of Breast Surgery.* Mosby; 2009:267-278.

5. The A. Anastrozole alone or in combination with tamoxifen versus tamoxifen alone for adjuvant treatment of postmenopausal women with early breast cancer: first results of the ATAC randomised trial. *The Lancet.* 2002;359(9324):2131-2139.

6. Mincey BA, Duh MS, Thomas SK, et al. Risk of cancer treatment-associated bone loss and fractures among women with breast cancer receiving aromatase inhibitors. *Clinical breast cancer.* 2006;7(2):127-132.

7. Crew KD, Greenlee H, Capodice J, et al. Prevalence of joint symptoms in postmenopausal women taking aromatase inhibitors for early-stage breast cancer. *Journal of Clinical Oncology.* 2007;25(25):3877-3883.

8. Bender CM, Sereika SM, Brufsky AM, et al. Memory impairments with adjuvant anastrozole versus tamoxifen in women with early-stage breast cancer. *Menopause (New York, NY).* 2007;14(6):995.

9. Cella D, Fallowfield L, Barker P, Cuzick J, Locker G, Howell A. Quality of life of postmenopausal women in the ATAC ("Arimidex", tamoxifen, alone or in combination) trial after completion of 5 years' adjuvant treatment for early breast cancer. *Breast cancer research and treatment.* 2006;100(3):273-284.

10. Breckenridge LM, Bruns GL, Todd BL, Feuerstein M. Cognitive limitations associated with tamoxifen and aromatase inhibitors in employed breast cancer survivors. *Psycho-Oncology.* 2012;21(1):43-53.

11. So WK, Marsh G, Ling W, et al. The symptom cluster of fatigue, pain, anxiety, and depression and the effect on the quality of life of women receiving treatment for breast cancer: a multicenter study. Paper presented at: Oncology nursing forum2009.

12. Jacobson E. Progressive muscle relaxation. *Interview Behaviour" Journal of Abnormal Psy-University of Chicago Piess, Chicago chology.* 1938;75(1):18.

13. Bell JA, Saltikov JB. Mitchell's relaxation technique: Is it effective? *Physiotherapy.* 2000;86(9):473-478.

14. Bernstein DA, CARLSON CR, SCHMIDT JE. Progressive relaxation. *Stress Management.* 1973:88.

15. Otman A, Köse N. Basic principles and methods in exercise therapy. *Meteksan AŞ.* 2006:21-51.

16. GALANTINO, Mary Lou, et al. Impact of yoga on functional outcomes in breast cancer survivors with aromatase inhibitor-associated arthralgias. Integrative Cancer Therapies, 2012, 11.4: 313-320.

17. Dicle A, Karayurt Ö, Dirimese EJPMN. Validation of the Turkish version of the Brief Pain Inventory in surgery patients. 2009;10(2):107-113. e102.

18. Cleeland C, Ryan KJA, Academy of Medicine, Singapore. Pain assessment: global use of the Brief Pain Inventory. 1994.

19. Fallowfield LJ, Leaity SK, Howell A, Benson S, Cella DJBcr, treatment. Assessment of quality of life in women undergoing hormonal therapy for breast cancer: validation of an endocrine symptom subscale for the FACT-B. 1999;55(2):187-197.

20. Aydemir OJTPD. Validity and reliability of hospital anxiety and depression scale Turkish form. 1997;8:187-280.

**Annex 1.**

27.03.2019

**Istanbul Demiroglu Science University**

**Chairmanship of the Clinical Research Ethics Committee**

Uzm. Physiotherapist Umut Bahçacı has been approved to conduct the non-interventional clinical research titled "**The effects of progressive relaxation exercises in breast cancer patients using aromatase inhibitors**" in the Medical Oncology Clinic of our institution.

Gayrettepe Florence Nightingale Hospital

Medical Director

Dr Ozay Unal

**Appendix 2.**

**INFORMED CONSENT FORM FOR RESEARCH STUDY**

The name of our research is 'The effects of progressive relaxation exercises in breast cancer patients using aromatase inhibitors'. If you wish to participate in the study after reading and understanding this information, please sign the form.

If you agree to participate in the study, your demographic information will be taken by the responsible researcher Assoc. Prof. Dr. Zeynep Erdoğan İyigün and your pain assessment will be performed. Then, assessments used to measure your quality of life, anxiety and depression will be applied. As a result of the evaluation, if you are deemed appropriate by Assoc. Fzt. Umut Bahçacı supervision, you will be included in the progressive relaxation exercises programme. Relaxation exercises will last for 6 weeks and at the end of this period, your second evaluations will be made. Your evaluation records can be used in the education of students studying in the field of health or in scientific publications without revealing your identity. Other than that, these records will not be used and will not be given to others.

In the study; the names of the volunteers will not be used and only the results will be published statistically in the form of a scientific article. Health authorities, the Ministry, the Ethics Committee can access the records of the volunteers when necessary, but this information will be kept confidential. Volunteers will be informed about the results of the study if they wish, and they have the right to leave the study at any time. Volunteers will not receive any compensation or additional payment for this study even if they leave the study. No additional treatment will be given during or after the study.

Risks that may occur during the assessments: the assessments to be carried out within the scope of the study and the programme to be implemented do not involve any risks.

**Participant's Declaration**

I was informed by Assoc. Prof. Dr. Zeynep Erdoğan İyigün that a research would be conducted to investigate "the effects of progressive relaxation exercises in breast cancer patients using aromatase inhibitors" and I was given the above information about this research. After this information, I was invited as a 'participant' in such a research.

If I participate in this research, I believe that the confidentiality of my personal information, which should remain between me and the doctor, will be treated with great care and respect during this research. I have been given sufficient confidence that my personal information will be carefully protected during the use of the research results for educational and scientific purposes.

I have read all the explanations in the Informed Voluntary Consent Form. Written and verbal explanations about the research, the subject and purpose of which are described above, were given to me by the researcher named below. I know that I am participating in the research voluntarily, that I can leave the research at any time with or without justification, and that I can be excluded from the research by the researcher regardless of my own will.

I have understood all the explanations given to me in detail. I have voluntarily decided to take part in this research on my own. I accept the invitation with great pleasure and willingness.

I will be given a copy of this signed form.

**I agree to participate in the said research voluntarily, without any pressure or coercion**

**I accept I do not accept**

**The information obtained for this study will be used in another study to be conducted in the future.**

**I accept I do not accept**

**Participant**

First name, last name:

Address:

Tel:

Signature:

**Interview witness**

First name, last name:

Address:

Tel.

Signature:

**The worker who interviewed the participant**

First name, last name:

Address:

Tel.

Signature:

Date: Tel:

**Annex 3.**

**Evaluation Form**

EVALUATION OF ARTERIAL INVOLVEMENT IN BREAST CA PATIENTS ON AROMATASE INHIBITORS

NAME:

AGE:

SIZE: WEIGHT: VKI:

WAIST CIRCUMFERENCE:

MARITAL STATUS

PROFESSION:

EDUCATION STATUS:

DOMINANT LIMB

DATE OF DIAGNOSIS:

OPERATION TIME:

OPERATION SIDE:

OPERATION TYPE:

CONTRAINDICATIONS

CT (AGENTS):

TAKSAN

TMX

RT:

AROMOTASE INH. A.K.A:

DURATION OF USE

TEA: COFFEE:

ALCOHOL: CIGARETTE:
