# Supplementary material for: Progressive relaxation training in patients with breast cancer receiving aromatase inhibitor therapy-randomized controlled trial
Source: PLoS One. 2024 Apr 18;19(4):e0301020. doi: 10.1371/journal.pone.0301020 (PMC11025930; doi:10.1371/journal.pone.0301020)
Supplement: Attachment — Submitted filename: Response to Reviewers.docx [file pone.0301020.s004.docx]

**Progressive Relaxation Training in Patients with Breast Cancer Receiving Aromatase Inhibitor Therapy-Randomized Controlled Trial**

**ID number= PONE-D-23-36160**

**Response to Reviewers**

The authors express gratitude towards the reviewers for their constructive comments. The paper has significantly improved due to the suggested changes. The authors are now going to address each of the comments made by the reviewers. The comments are presented in red, and the authors' responses are in black. We have highlighted the corresponding changes in our revised manuscript titled 'Revised manuscript with Track Changes'.

**Journal Requirements:**

1) Please ensure that your manuscript meets PLOS ONE's style requirements, including those for file naming. The PLOS ONE style templates can be found at
   <https://journals.plos.org/plosone/s/file?id=wjVg/PLOSOne_formatting_sample_main_body.pdf> and 
   <https://journals.plos.org/plosone/s/file?id=ba62/PLOSOne_formatting_sample_title_authors_affiliations.pdf>.

**Authors’ Response:** **After reviewing the comments of reviewers, we have checked the manuscript again and made necessary changes to ensure it met PLOS ONE's style requirements.**

2) Please include captions for your Supporting Information files at the end of your manuscript, and update any in-text citations to match accordingly. Please see our Supporting Information guidelines for more information: <http://journals.plos.org/plosone/s/supporting-information>.

**Authors’ Response: Our supporting information file labelled ‘S1 CONSORT checklist’ has been included at the end of the manuscript before ‘References’.**

**Additional Editor Comments:**
This is an interesting article and hypothesis, and the results are food for thought. Congratulations for the work done.
Still the comments made by the reviewers are valid and need to be adressed and responded to in a satisfactory way, before going further in the process.

**Authors’ Response: We have revised it carefully and are pleased to present the updated version of our work. We have made substantial changes with the constructive feedback of reviewers. All necessary responses are clearly stated below.**

**Thank you and all reviewers for reading and effort to make our work better.**

**Reviewer #1:  A two-arm randomized controlled clinical trial was conducted which aimed to assess the efficacy of Progressive Relaxation Exercises on the side effects of aromatase inhibitors in patients with breast cancer. The intervention group reported significantly less pain severity and pain interference than the controls. No differences in quality of life and emotional status were found.**

**Authors’ Response: The authors appreciate the reviewer's thorough analysis of the present work and valuable feedback. Necessary changes have been made in line with comments and requests and are based on scientific data.**

Minor revisions:
**1- P-values never equal zero; express small p-values as < 0.001.**

**Authors’ Response: We detected two p values equal to zero. And we changed these by expressing them as p<0.001. Changes are highlighted in the draft.**

**2- Line 122: Provide full details of the sample size calculation. The power calculation should include: (1) the estimated outcomes in each group; (2) the α (type I) error level; (3) the statistical power (or the β (type II) error level); (4) the target sample size (5) statistical testing method and (6) for continuous outcomes, the standard deviation of the measurements.**

“**Authors’ Response: This section has been reviewed carefully. The power and error levels were clearly stated in the sample size calculation. The text explicitly stated the estimated outcomes, target sample size, mean, and standard deviation of the parameter used. The statistical test method was mentioned in the "Statistical analysis" section. All changes have been highlighted in the text and depicted below:**

“The mean and standard deviation data of the QoL parameter [mean (SD): 89,33(20,18) and 106,05(17,02) respectively] were used. The target sample size was determined with the aim of recruiting at least 20 participants per group, based on a power of 80% and a confidence interval of 95%, with the effect size 0,88. Including estimated dropout rate of 30%, it is planned to recruit 26 patients in each group.”

**3- Line 140: Indicate if block randomization was used. If so, specify the block size.**

**Authors’ Response: Our study did not use block randomization. Instead, we used simple randomization with a computer-generated random sequence of numbers (from the site:** [**www.random.org**](http://www.random.org)**) and allocated them accordingly. We reviewed the randomization statement in the manuscript and improved our expressions for clarity. We have made necessary changes and highlighted them in the manuscript as shown below:**

**“**Participants were allocated randomly to either the study group (n=27) or the control group (n=27) in a 1:1 ratio, utilizing a list of computer-generated random sequence numbers. The computer-generated list was used to check the last two digits of the participants’ protocol number and determine the group. After this determination, the next participant was placed in the other group.**”**

**4- Line 212: For improved clarity, replace “analyzed” with “summarized.”**

**Authors’ Response: The word 'summarized' was used instead of 'analyzed' and highlighted in the 'Statistical analysis' section.**

**5- Table 3: When data is not normally distributed, it is customary to summarize the results using median, first and third quartiles.**

**Authors’ Response: We reviewed the statistical analysis of the study. To comply with non-parametric analysis, we have added median and quartile (25% and 75%) data in the table, as suggested. All changes have been highlighted in the table.**

**Thank you for the time and effort you put in to improve our work.**

**Reviewer #2:** **This -to my knowledge-is the first study testing 'Progressive Relaxation Exercises' in patients receiving an oral aromatase inhibitor in early breast cancer. The study is positive for some endpoints (Pain); the authors do caution their conclusions saying that longer follow-up is needed and that PRE has no effect on other studied endpoint.**

**Authors’ Response: The authors appreciate the reviewer's thorough analysis of the present work and valuable feedback. Necessary changes have been made as commented and requested and are based on scientific data.**

**- Please use the official term 'AIMSS' throughout the manuscript as this is the name given to this well-known problem (aromatase inhibitor musculoskeletal syndrome)**

**Authors’ Response: We defined all the terms, referring to AI-related pain or describing AI-related musculoskeletal problems, as AI-MSS throughout the manuscript. All alterations have been highlighted in the manuscript.**

**- Please describe the pathophysiology of AI-MSS (refering to fluid retention in the joints as studies by MR imaging and ultrasound); Morales et al. J Clin Oncol 2008**

**Authors’ Response: We would like to thank you for this valuable suggestion. We read the related article and searched for other related articles too. And we restructured the relevant part of the manuscript as shown below:**

“Almost 50% of patients receiving AI experience arthralgia, particularly as part of AI-induced musculoskeletal syndrome (AI-MSS) [6]. Some studies have explained the mechanism of arthralgia as being caused by estrogen deficiency, which can lead to bone deficiency and arthritis [7]. Additionally, MRI findings have revealed the pathophysiology of AI-MSS, which is associated with tenosynovial changes such as tenosynovial fluid accumulation and edema in the subcutaneous tissue that may cause pain in patients receiving AI [8, 9].”

**- The authors report on mean and SD; What clinicians do want to know is whether women (in one group) with more pain at baseline were more likely to respond to PRE/no-PRE than those with less pain at baseline.**

**Authors’ Response: We thank the reviewer for the contribution. In this study, our primary aim was to analyze the mean scores of the changes in pain between baseline and 6 weeks after the intervention. Therefore, M and SD values were given to show this change. Clinicians should be aware that it is meaningful to compare changes between groups. As a result, there was an increase in the scores of this change in pain assessment in the control group.**

**- Some minor edits to be corrected in the text**

**Authors’ Response: The entire manuscript has been re-edited. Relevant changes have been made based according the comments and highlighted in the text. Thank you very much for your contribution to the improvement of our work.**

**Reviewer #3: The authors, Bahcaci et al. present data from their RCT on “Progressive Relaxation Training in Patients with Breast Cancer Receiving Aromatase Inhibitor Therapy-Randomized Controlled Trial,” which addresses an important area of morbidity for a large proportion of breast cancer patients treated with AIs, however the small sample size and the lack of analytical rigor limit clinical interpretation of this data.**

**Authors’ Response: The authors appreciate the reviewer's thorough analysis and valuable feedback on the current study. As 'Progressive Relaxation Exercises' has not been done in any research paper for these sample, we used similar studies using yoga for the same group of patients to estimate the sample size. Therefore, although the sample size seems to be small, we request that the efforts of this study be taken into account, which showed good effect size in the G power analysis and effect size. We have also mentioned it in the manuscript as a limitation of the study. The analysis of the manuscript has been completely re-reviewed and necessary adjustments have been made based on the reviewers' comments to improve analytical rigor.**

Introduction: **Paragraph 1 is not needed, especially since mechanism of action is not well-described, it adds minimal value to the context. It is OK to open with Paragraph #2.**

**Authors’ Response: In line with the reviewers' suggestions, the first paragraph has been removed and the introduction has been contextualized by adding information about the mechanism of action in line with the suggestions. Introduction has been reorganized. All changes are highlighted in the manuscript.**

**For Paragraph 2 in addition to indication listed, it is important to expand on the magnitude of the problem that AI-related adverse events lead to discontinuation of therapy.**

**Authors’ Response: We thank to the reviewer for his contribution. We have researched the literature in line with the suggestion and added some mechanisms of AI-induced arthralgia and AI-induced musculoskeletal syndrome to the paragraph. We have expanded the pathophysiology underlying AI-associated adverse events and used the official term AIMSS (Aromatase Inhibitor Musculoskeletal Syndrome) in the manuscript as shown below:**

“Almost 50% of patients receiving AI experience arthralgia, particularly as part of AI-induced musculoskeletal syndrome (AI-MSS) [4]. The most common musculoskeletal adverse events associated with AI-MSS are bone loss and arthralgia, which are primarily caused by estrogen deficiency [5]. The mechanism behind AI-induced arthralgia has been defined in several ways [6]. One is that reduced estrogen levels increase pro-inflammatory cytokines such as IL-6 and IL-1 in articular cartilage cells, causing joint pain and swelling [7]. The other mechanism is that the aromatase and estrogen receptors are expressed in the brain and the spinal cord analgesic system. This contributes to significantly reduced pain thresholds. This contributes to significantly reduced pain thresholds [8]. Additionally, MRI findings have revealed the pathophysiology of AI-MSS, which is associated with tenosynovial changes such as tenosynovial fluid accumulation and edema in the subcutaneous tissue that may cause pain in patients receiving AI [9, 10]”

**Paragraph #3 - be specific which adverse effects are mitigated by exercise interventions and what type of exercise interventions. The current description seems very broad and again non-specific.**

**Authors’ Response: We re-built the paragraph with enhancing information on adverse effects that are mitigated by exercise intervention. And we added some more data about the type of exercises as depicted below:**

“Besides pharmacological approaches, other interventions to reduce AI side effects include acupuncture, nutritional supplementation, relaxation techniques and physical exercise. Exercise interventions have been recommended as a means of alleviating the side effects of AI in patients with breast cancer (BC). These interventions include walking, aquatic exercise, strength training, bench press, leg press, seated row, and others [6, 16]. Aerobic, resistive or combined exercises are commonly used to improve the quality of life in BC patients undergoing AI treatment [17, 18]. These exercise interventions are mostly effective on reduction in pain and stiffness, improving in muscle strength, quality of life and pain threshold [16]. Only two studies, one involving yoga and the other Thai-chi, have examined the effectiveness of relaxation techniques on AI associated arthralgia. [19]. The non-utilization of direct relaxation exercises to reduce AI side effects can be considered as a gap. However, there is inadequate evidence regarding the impact of relaxation techniques on treatment-related side effects in breast cancer patients.”

**Paragraph 4 mixes data on muscle relaxation and exercise interventions. It is important to define the components on muscle relaxation exercises and the effect sizes seen in other populations to establish scientific plausibility that PRE would work in AI-induced arthralgias**

**Authors’ Response:** **In line with your suggestions, the components of progressive muscle relaxation are now briefly mentioned in the paragraph. Detailed instructions of PRE are given in Table 1. We also included the effects of PRE on other events in breast cancer patients and lung cancer patients. All necessary changes are highlighted in the text and depicted below:**

**“**PRE is a technique used in various studies to relax the whole body. It works by maximum contraction and relaxation of different muscle groups, sometimes accompanied by deep breathing [20, 21]. PRE is a widely used method with many current modifications for various health conditions [22, 23]. Physiological, perceptual, and behavioral positive findings of muscle relaxation were well-defined [21]. PRE is effective in breast cancer patients, providing improvements in upper limb function and reducing anxiety, as well as reducing the severity of chemotherapy symptoms [24-26]. On the other hand, PRE appears to be effective in managing symptoms and increasing self-efficacy in lung cancer patients [27]. Considering the types of exercises, it can be inferred that patients may find it easier to perform PRE and therefore prefer it.”

Methods:

**The details of the sample size calculation are inadequate – the authors should define what effect size they expected to see with a power of 80% and alpha of 0.05. A sample size of 20 per arm is a very small number for meaningful effect size**

**Authors’ Response: This section has been reviewed carefully with your valuable comments. Power (80%), confidence interval (95%) and effect (0,88) of the sample size calculation are now clearly defined. The text explicitly stated the estimated outcomes, target sample size, mean, and standard deviation of the parameter used. The statistical test method of the sample size was mentioned in the "Statistical analysis" section. All changes have been highlighted in the text and depicted below:**

“The mean and standard deviation data of the QoL parameter [mean (SD): 89,33(20,18) and 106,05(17,02) respectively] were used. The target sample size was determined with the aim of recruiting at least 20 participants per group, based on a power of 80% and a confidence interval of 95%, with the effect size 0,88. Including estimated dropout rate of 30%, it is planned to recruit 26 patients in each group.”

**The study was analysed with a total of 44 participants, with 22 participants per group. The sample size was determined using a similar study with an effect size of 0.88. We are sorry for any concerns regarding the sample size. It took us 1.5 years to complete the PRE sessions and reach this number. We encountered several challenges during implementation in finding and retaining participants (for both groups). Recruitment difficulties, such as those found in a single-centred study, patient non-adherence, and cancer psychology, can affect the sample size that has been reached. However, we have included a statement in the 'discussion' section suggesting the need for similar studies with a larger sample size.**

**Was the PRE implemented to the study participants as a group – in which case peer support will be a big confounder? I.e. did scores in pain and QALY improved due to the community/support aspect of the group and not necessarily the intervention in and of itself? Should the analysis be intent to treat – patients excluded if they missed 10% of sessions? How did you establish fidelity of the at home sessions?**

**Authors’ Response: Peer and community support are important factors to consider in this study. While it can positively impact patients, it should also be considered as a part of the intervention. Many exercise studies include supportive elements such as peer support, manager support, facilities in the exercise room, and support from other healthcare professionals, as well as restrictive elements such as time spent on exercise and transportation, financial situations, and despair due to illness. These factors are inherent in exercise studies. Therefore, we included it as one of the limitations of the study. This has been emphasized in the limitation section of text and depicted below:**

“In contrast, the peer support in the intervention group, which can affect intervention results positively or negatively, can be considered a limitation.”

**We have 4 patients who missed the 10% of session (more than 3 days in total) in study group. These patients weren’t included in the analysis. The analysis was conducted on patients who didn't miss more than 3 days of sessions. Additionally, an equal number of participants (five) from each group were excluded during the follow-up period of the study. Thus, the process of flow proceeded almost similarly for both groups. Please see the Fig 1. Consort Flow Diagram.**

**For the fidelity of the at home sessions were made by phone calls in every exercise day as we mentioned in Method section. We encouraged participants also in the supervise sessions. Each exercise item was queried on phone calls and participants were asked to complete their exercise diary. We devoted a considerable amount of time to encouraging and ensuring that participants attended all sessions.**

**All exercises were performed by the same physiotherapist who is experienced in oncological rehabilitation. The exercise room, temperature, lighting, and ventilation were consistent throughout the study. The intervention procedure was also consistent throughout the study process. We endeavoured to maintain equal conditions for each participant.**

**Not sure if statistical approach evaluating means is the appropriate approach, Eg. for table 3, authors assess mean in comparing scores on outcome instruments, but median and non-parametric analysis as they listed would have been most appropriate.**

**Authors’ Response: The statistical approaches are carefully described in the relevant section of the paper. We have reviewed the statistical analysis and re-consulted with our statistician to ensure the appropriateness for this study. To comply with the nonparametric analysis, we have added median and quartile (25% and 75%) data to the table as suggested. All changes to the table are highlighted in the table.**

Results: **Baseline characteristics should have also included stage of breast cancer as well as number of years on AI.**

**Authors’ Response: The table has been updated to include data on breast cancer stage. This information is highlighted in the table. According to the inclusion criteria, we selected participants who had received AI treatment for more than 6 months. According to the literature, the median time from initiation of AI treatment to the onset of musculoskeletal symptoms is 1.6 months (range 0.4-10 months) and symptoms tend to peak at ∼6 months*. The appropriateness of this 6-month duration of AI was discussed with our authors, who are medical oncologists and surgeons, and we started the study by defining our accepted inclusion criteria accordingly. Therefore, no record of the duration of AI use was kept afterwards. We believe that it would be important to take into account that we have created the 6-month usage information with the suggestions of our oncologists in line with the sources mentioned below. References to the relevant articles are below:**

***** 1) Niravath P. Aromatase inhibitor-induced arthralgia: a review. Annals of Oncology. 2013 Jun 1;24(6):1443-9.

2) Henry NL, Giles JT, Ang D, Mohan M, Dadabhoy D, Robarge J, Hayden J, Lemler S, Shahverdi K, Powers P, Li L. Prospective characterization of musculoskeletal symptoms in early stage breast cancer patients treated with aromatase inhibitors. Breast cancer research and treatment. 2008 Sep;111:365-72.

**Table 3 – baseline scores for Table 3 for BPI-PS are higher for control group than for study group, and especially since the significance level is based on 1-point differences in report scores, I think this should be discussed as a limitation in interpreting effect size. Perhaps the model should have adjusted for baseline level of pain assessment. Results should also be interpreted in terms of what is a clinically significant difference between the groups – effect size between groups is not significant for any of the instruments. The instruments were described as assessing scores of 3-4 as mild, so is this study result clinically significant if the mean score @ 6 weeks for post groups fall within the mild range? In some cases, below the mild scale (<3).**

**Authors’ Response: Thank you for your contribution. Looking again, we found that BPI scores at baseline were higher in the study group than in the control group. We appreciate this comment about 'baseline scores'. However, as this was the first study, we were primarily interested in seeing the effect on pain. An explanation of this is therefore included in the limitations section of the study, as shown below:**

“The other limitation is that the baseline BPI scores were higher in the study group than in the control group. This is noteworthy because it is important that even a 1-point difference can affect significance. However, the effect size between groups is low in both 'BPI-PS' and 'BPI-PI' scores.”

**The study's effect sizes are in the small and medium range due to the small sample size. The aim was to demonstrate the impact of relaxation exercises on the estimated sample size, even if the effect size was small. However, it is suggested in the text that future studies should be conducted with a larger sample size as depicted below:**

“Long-term follow-up procedures with an increased sample size could be meaningful for future studies to ensure patient compliance with complementary therapies.”

**Our goal was to analyse the mean scores of the change between baseline and after the 6-week intervention. Table 3 highlights these scores. However, the categorization of pain was not utilized in this study. According to The BPI User Guide*, many articles on BPI in cancer use mean scores. It is also established that higher BPI scores are linked to higher Numeric Rating Scale scores^1^. However, we intend to use these descriptive scores to help inform our future studies. In this context, we have removed the statement “Scores of 3-4 are defined as mild, 5-7 as moderate and 8-10 as severe pain in this scale” in the BPI explanation and replaced it with the statement “Higher scores are associated with higher pain” with adding the relevant reference. All changes are highlighted in the text.**

* <https://www.mdanderson.org/content/dam/mdanderson/documents/Departments-and-Divisions/Symptom-Research/BPI_UserGuide.pdf>

1) Dana D Im, Guruprasad D Jambaulikar, Anna Kikut, Jasmine Gale, Scott G Weiner, Brief Pain Inventory–Short Form: A New Method for Assessing Pain in the Emergency Department, *Pain Medicine*, Volume 21, Issue 12, December 2020, Pages 3263–3269.

Discussion: **The limitation section should be further expanded not a cursory statement that the sample size was small. Fidelity to home sessions should also be discussed. Authors should also discuss potential mediators of the effect noted. This should be an ITT analysis, is there a reason why the authors didn’t consider this?**

**Authors’ Response: We have improved our limitations section with the valuable comments. A statement has also been added about the fidelity of home sessions after the limitations section. All changes, for the limitations and the statement, have been highlighted in the text and depicted below:**

“In contrast, the peer support in the intervention group, which can affect intervention results positively or negatively, can be considered a limitation. The other limitation is that the baseline BPI scores were higher in the study group than in the control group. This is noteworthy because it is important that even a 1-point difference can affect significance. However, the effect size between groups is low in both 'BPI-PS' and 'BPI-PI' scores. Long-term follow-up procedures with an increased sample size could be meaningful for future studies to ensure patient compliance with complementary therapies. Although telephone reminders and exercise diary keeping can improve fidelity to home exercises in this study, the use of fully supervised relaxation exercises will give more meaningful results.”

**The study's dropout rate is 18%, with 54 participants randomized and 44 analyzed. This rate falls within an acceptable range^1^. Although the same number of participants were lost during the follow-up periods in both groups, it cannot be concluded that the estimates are biased or unbiased^2^. However, it can be stated that the reasons for dropouts were similar. Participants were lost during the follow-up period due to personal reasons related to their individual desires. In addition, the method of analysis was the same for both groups. So, it is believed that the potential mediators that could affect the intention-to-treat analysis are within acceptable limits. Relevant references are shown below:**

1) Wood AM, White IR, Thompson SG. Are missing outcome data adequately handled? A review of published randomized controlled trials in major medical journals. *Clinical Trials*. 2004;1(4):368-376. doi:[10.1191/1740774504cn032oa](https://doi.org/10.1191/1740774504cn032oa)
2) Bell ML, Kenward MG, Fairclough DL, Horton NJ. Differential dropout and bias in randomised controlled trials: when it matters and when it may not. BMJ. 2013 Jan 21;346:e8668. doi: 10.1136/bmj.e8668. PMID: 23338004; PMCID: PMC4688419.

Minor comments:
**In the abstract, line 44 has an incomplete p value listed – 0.000.**

**Authors’ Response: The p value is corrected as p<0,001 and highlighted in the text.**

**The authors thank the reviewer for their time and effort in improving our work.**
