# Supplementary material for: Progressive relaxation training in patients with breast cancer receiving aromatase inhibitor therapy-randomized controlled trial
Source: PLoS One. 2024 Apr 18;19(4):e0301020. doi: 10.1371/journal.pone.0301020 (PMC11025930; doi:10.1371/journal.pone.0301020)
Supplement: Attachment — Submitted filename: Response to Reviewers.docx [file pone.0301020.s005.docx]

**Progressive Relaxation Training in Patients with Breast Cancer Receiving Aromatase Inhibitor Therapy-Randomized Controlled Trial**

**ID number= PONE-D-23-36160**

**Response to Reviewers**

The authors would like to thank the reviewer for the comment. The comments are presented in red, and the authors' responses are in black. We have highlighted the corresponding changes in our revised manuscript titled 'Revised manuscript with Track Changes'.

**Journal Requirements:**

1) Please review your reference list to ensure that it is complete and correct. If you have cited papers that have been retracted, please include the rationale for doing so in the manuscript text, or remove these references and replace them with relevant current references. Any changes to the reference list should be mentioned in the rebuttal letter that accompanies your revised manuscript. If you need to cite a retracted article, indicate the article’s retracted status in the References list and also include a citation and full reference for the retraction notice.

**Authors’ Response:** **We have checked reference list and made necessary changes to ensure it met PLOS ONE's style (Vancouver) requirements.**

**Additional Editor Comments:**
Dear author
Thank you for the revisions made.
Kindly attend to the minor revision suggestion of Reviewer 1, then we can go on and accept the article.

**Authors’ Response: The authors would like to thank the editor for their effort and interest. The necessary minor revision suggestion of Reviewer 1 has been incorporated.**

**Thank you and all reviewers for reading and effort to make our work better.**

**Reviewer #1:  Minor revisions:
Table 2: In addition to frequencies, add the corresponding percentages.**

**Authors’ Response: The authors kindly thank the reviewer for their feedback. The percentages of frequencies are now included in Table 2.**
